# The Roles of S100A4 and the EGF/EGFR Signaling Axis in Pulmonary Hypertension with Right Ventricular Hypertrophy

**DOI:** 10.3390/biology11010118

**Published:** 2022-01-12

**Authors:** Maria Laggner, Philipp Hacker, Felicitas Oberndorfer, Jonas Bauer, Thomas Raunegger, Christian Gerges, Tamás Szerafin, Jürgen Thanner, Irene Lang, Nika Skoro-Sajer, Hendrik Jan Ankersmit, Bernhard Moser

**Affiliations:** 1 Department of Thoracic Surgery, Medical University of Vienna, 1090 Vienna, Austria; maria.laggner@meduniwien.ac.at (M.L.); jonas.bauer@meduniwien.ac.at (J.B.); juergenthanner@gmx.at (J.T.); hendrik.ankersmit@meduniwien.ac.at (H.J.A.); 2 Applied Immunology Laboratory, Medical University of Vienna, 1090 Vienna, Austria; 3 Department of Oral and Maxillofacial Surgery, University Hospital St. Pölten, 3100 St. Pölten, Austria; Philipp11@gmx.at; 4 Department of Pathology, Medical University of Vienna, 1090 Vienna, Austria; felicitas.oberndorfer@meduniwien.ac.at; 5 Division of Internal Medicine and Cardiology, Klinikum Klagenfurt am Wörthersee, 9020 Klagenfurt, Austria; raunegger.thomas@gmail.com; 6 Department of Medicine II, Division of Cardiology, Medical University of Vienna, 1090 Vienna, Austria; christian.gerges@meduniwien.ac.at (C.G.); irene.lang@meduniwien.ac.at (I.L.); nika.skoro-sajer@meduniwien.ac.at (N.S.-S.); 7 Department of Cardiac Surgery, Faculty of Medicine, University of Debrecen, 4032 Debrecen, Hungary; szerafin@med.unideb.hu

**Keywords:** pulmonary hypertension, chronic thromboembolic pulmonary hypertension, idiopathic pulmonary arterial hypertension, S100A4, epidermal growth factor, epidermal growth factor receptor, endothelial-to-mesenchymal transition, atrial valve stenosis

## Abstract

**Simple Summary:**

Pulmonary hypertension (PH) is a condition characterized by increased pulmonary arterial pressure. PH can further lead to right ventricular hypertrophy (RVH) and, consequently, heart failure. Endothelial-to-mesenchymal transition (EndMT) was identified as a key process in PH pathology. Nonetheless, the exact systemic and local levels of EndMT factors have not been comprehensively studied so far. Here, we quantified S100A4, epidermal growth factor (EGF), and EGF receptor (EGFR) in serum and tissue samples of two classes of PH with RVH. Patients with left heart disease and healthy volunteers served as controls. Serum S100A4 was decreased in the PH groups investigated, while EGF levels were increased in one PH cohort. EGFR was diminished in all groups compared to healthy controls. Surgical treatment of PH showed no effect on systemic EndMT marker levels. Furthermore, we observed a positive correlation between advanced PH stages and S100A4. Together, these findings help to deepen our understanding of the complex molecular events contributing to PH pathology and disease progression.

**Abstract:**

Pulmonary hypertension (PH) is characterized by increased pulmonary arterial pressure caused by the accumulation of mesenchymal-like cells in the pulmonary vasculature. PH can lead to right ventricular hypertrophy (RVH) and, ultimately, heart failure and death. In PH etiology, endothelial-to-mesenchymal transition (EndMT) has emerged as a critical process governing the conversion of endothelial cells into mesenchymal cells, and S100A4, EGF, and EGFR are implicated in EndMT. However, a potential role of S100A4, EGF, and EGFR in PH has to date not been elucidated. We therefore quantified S100A4, EGF, and EGFR in patients suffering from chronic thromboembolic pulmonary hypertension (CTEPH) and idiopathic pulmonary arterial hypertension (iPAH). To determine specificity for unilateral heart disease, the EndMT biomarker signature was further compared between PH patients presenting with RVH and patients suffering from aortic valve stenosis (AVS) with left ventricular hypertrophy. Reduced S100A4 concentrations were found in CTEPH and iPAH patients with RVH. Systemic EGF was increased in CTEPH but not in iPAH, while AVS patients displayed slightly diminished EGF levels. EGFR was downregulated in all patient groups when compared to healthy controls. Longitudinal data analysis revealed no effect of surgical therapies on EndMT markers. Pulmonary thrombo-endarterectomized samples were devoid of S100A4, while S100A4 tissue expression positively correlated with higher grades of Heath–Edwards histopathological lesions of iPAH-derived lung tissue. Histologically, EGFR was not detectable in CTEPH lungs or in iPAH lesions. Together, our data suggest an intricate role for S100A4 and EGF/EGFR in PH with right heart pathology.

## 1. Introduction

The 2015 consensus guidelines of the European Society of Cardiology (ESC) and the European Respiratory Society (ERS) define pulmonary hypertension (PH) as an increased mean pulmonary arterial pressure (mPAP) ≥ 25 mmHg at rest [1,2]. Recently, the definition of PH was newly defined with an mPAP threshold > 20 mmHg [3,4]. PH may be associated with different cardiovascular and pulmonary diseases. Hence, six distinct subgroups are recognized by ESC/ERS based on different causes and conditions of PH [5]. The first group represents pulmonary arterial hypertension (PAH), which can be elicited by various conditions, such as mutations of specific loci, certain drug treatments, congenital heart disease, liver disease, human immunodeficiency virus infection, or autoimmune diseases. The exact underlying cause, however, remains obscure in the prevailing majority of PAH cases. These cases are collectively referred to as idiopathic PAH (iPAH) [6]. The incidence of PAH was determined as six cases per million [7]. Even with clinical management, five-year survival rates of patients suffering from PAH are below 60% [8]. Characteristic histopathological changes of iPAH occur in distal pulmonary arteries smaller than 500 µm in diameter, and it is postulated that dysfunctional cells of the pulmonary vasculature are responsible for these alterations. A modified Heath–Edwards classification system is commonly used to grade histopathological changes observed in patients with iPAH, which typically include intimal hyperplasia, media hypertrophy, transmural inflammation, and thrombotic and characteristic plexiform lesions [9,10]. Continuous inflammation, vasoconstriction, and obstructive vascular remodeling contribute to increased pulmonary vascular resistance (PVR), which is propagated to the heart where it manifests as pressure overload to the right ventricle [11]. Clinical management of PAH usually depends on disease severity and is based on the response to treatment. Balloon atrial septostomy (BAS) and lung transplantation (LuTX) represent the gold standard surgical interventions of PAH.

Chronic thromboembolic pulmonary hypertension (CTEPH) represents a distinct subgroup in the ESC/ERS clinical classification of PH [5]. CTEPH is often triggered by unresolved pulmonary embolism (PE), though alternative underlying mechanisms have also been identified [12]. An estimated 0.1–9.1% of patients with symptomatic PE develop CTEPH within two years after diagnosis [13], though 25% cases of CTEPH have no history of PE [14]. In 2016, the incidence of CTEPH in Germany was reported to be 5.7 cases per million [15], though CTEPH is often underdiagnosed due to the non-specific symptoms of the disease. Five-year survival rates of inoperable CTEPH are below 70%. In stark contrast to iPAH, central, proximal pulmonary arteries are subjected to pathological restructuring. Similar arteriopathy as in PAH is observed in CTEPH disease pathology, including fibrosis, blood vessel thickening, and hyperproliferation [13]. Continued occlusion and ensuing vascular remodeling are followed by an increase in PVR and eventually lead to right heart failure [12]. The surgical gold standard technique is pulmonary endarterectomy (PEA), while balloon pulmonary angioplasty will be performed if patients are considered inoperable.

In spite of the different disease etiologies, progression of both iPAH and CTEPH is characterized by increased pressure overload to the right ventricle leading to right ventricular hypertrophy (RVH). Structural cardiac remodeling eventually causes progressive organ dysfunction, and heart failure represents the leading cause of death in PH patients [16]. Various factors have been identified to play a crucial role in the conversion of compensatory RV hypertrophy into decompensated RV failure, such as inflammation, neurohormones, oxidative stress, and ischemia. In contrast to PH, aortic valve stenosis (AVS) is a pathology with mechanical stress on the left ventricle, resulting in left ventricular hypertrophy. Cardiac hypertrophy in PH and AVS thus has different etiologies and represents a disease-specific tissue response to pressure overload.

Endothelial-to-mesenchymal transition (EndMT) is a process closely resembling epithelial-to-mesenchymal transition (EMT), which has been extensively studied in cancer progression and metastasis. During embryogenesis, EndMT is crucial for the development of pulmonary arteries [17] and contributes to morphogenesis of atrioventricular valves, semilunar valves, and the septum. Postnatally, EndMT is involved in the pathogenesis of vascular calcification, organ fibrosis, and PAH [18]. In PH-associated EndMT, endothelial cells of pulmonary arterioles transit into multipotent mesenchymal progenitor cells. Morphologically, endothelial cells lose their characteristic cobblestone structure and acquire a fibroblast-like shape. This reorganization is accompanied by a downregulation of endothelial markers with concomitant upregulation of characteristic mesenchymal pathways [19]. Different molecular mechanisms have been proposed to mediate EndMT, such as TGFβR-dependent signaling via TGFβ and BMP, canonical and non-canonical Wnt signaling, TNFα and IL1β inflammatory signaling, Notch signaling, or hypoxia-induced signaling. Furthermore, epidermal growth factor (EGF) and EGF receptor (EGFR) signaling promote EndMT in endothelial cells [20]. In situ evidence for EndMT in PH has been reported previously [21]. Several transcriptional factors (TFs) are implicated in the EndMT process, such as TWIST and Snail Family Transcriptional Repressor 1 and 2 (SNAI1 and SNAI2) [22], and these TFs have already been linked to PH pathology. Twist1 phosphorylation is required for hypoxia-induced PH [23], and SNAI1 and SNAI2 upregulation was found responsible for lung endothelial cells of iPAH patients acquiring a mesenchymal phenotype [24]. These TFs are known to repress the expression of endothelial cell markers, such as vascular endothelial cadherin [25] and CD31 [26], thereby contributing to the loss of an endothelial phenotype. In spite of these previous studies, systemic and local levels of additional EndMT markers in different types of PH have not yet been comprehensively studied.

S100 Calcium Binding Protein A4 (S100A4) belongs to the S100 calcium-binding protein family and is highly expressed in the bone marrow, lymphoid tissues, and lungs. Extracellular S100A4 can exert cytokine-like functions and acts synergistically with various ligands to engage different receptors, such as receptor for advanced glycation end-products (RAGE) and epidermal growth factor receptor (EGFR) [27]. Several co-ligands of S100A4 have been reported for EGFR activation, including EGF [28], heparin-binding EGF, and amphiregulin [27]. Previously, our group reported a role for RAGE signaling in PH [29], and RAGE exacerbated PH and has been proposed as a biomarker for disease severity [30]. Though S100A4 [31] and the EGF/EGFR axis have been implicated in EndMT and EndMT was found to contribute to PH, the potential role of S100A4 and EGF/EGFR in PH so far remains elusive. To this end, we sought to investigate EndMT biomarkers in patients with PH. First, we aimed to quantify serum concentrations of the EndMT molecules S100A4, EGF, and EGFR in iPAH and CTEPH. In order to test the specificity of EndMT markers for PH with pressure overload to the right ventricle, patients with left ventricular strain due to aortic valve stenosis were selected for comparison. Secondly, we aimed to determine a potential effect of surgical therapies for iPAH, CTEPH, and AVS on serum EndMT biomarkers. Lastly, we investigated tissue expressions of S100A4 and EGFR in endarterectomized specimens of CTEPH and pulmonary parenchyma of explanted lungs.

## 2. Materials and Methods

### 2.1. Ethics Statement

This study was approved by the institutional review board on human research of the Medical University of Vienna (Vienna, Austria; ethic vote # 1805/2013) and of the University of Debrecen (Debrecen, Hungary, ethic vote # 1331/2013). This study was performed in accordance with the Declaration of Helsinki and applicable local regulations. Written informed consent was obtained from all participants.

AVS aortic valve stenosis; CTEPH chronic thromboembolic pulmonary hypertension; EGF epidermal growth factor; EGFR epidermal growth factor receptor; ELISA enzyme-linked, immunosorbent assay; IHC immunohistochemistry; iPAH idiopathic pulmonary arterial hypertension; LuTX lung transplantation; LVH left ventricular hypertrophy; PEA pulmonary endarterectomy; RVH right ventricular hypertrophy; S100A4 S100 Calcium Binding Protein A4.

### 2.2. Study Design and Groups

**Figure 1:**
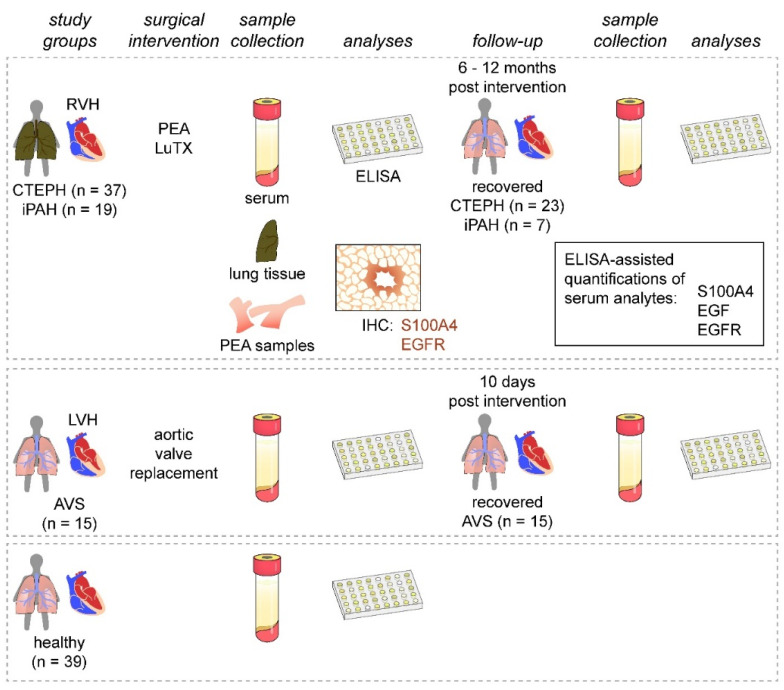
Study design. Patients presenting with PH (CTEPH and iPAH) and RVH were enrolled and compared to patients with AVS and LVH. Healthy volunteers served as controls. Serum samples were obtained from all study groups and during patient follow-up visits after surgical interventions. Serum samples were analyzed for S100A4, EGF, and EGFR. Endarterectomized specimens and iPAH lung tissues were used for immunohistochemical assessment of S100A4 and EGFR expressions.

This study was designed as a prospective, longitudinal, cross-sectional cohort study performed by the Department of Thoracic Surgery, Medical University of Vienna, Vienna, Austria and the Department of Cardiac Surgery, University of Debrecen, Debrecen, Hungary, between November 2013 and November 2016 (Figure 1). We enrolled 37 patients with CTEPH undergoing PEA and 19 patients with iPAH receiving lung transplantation (LuTX) at the Department of Thoracic Surgery, Medical University of Vienna. Guidelines used for patient diagnosis were established previously [2,32]. Additionally, 15 patients suffering from severe AVS undergoing aortic valve replacement at the Department of Cardiac Surgery, University of Debrecen were included. We further recruited 39 healthy volunteers with no prior evidence of pulmonary hypertension, cardiac abnormalities, infectious diseases, or malignancies. As patient groups displayed great differences in basic demographic characteristics (age and sex), we allocated age- and sex-matched subgroups of healthy volunteers to each respective patient cohort (Table 1, Table 2 and Table 3).

### 2.3. Serum Sample Collection

Preoperative serum samples were collected one day before surgery. Postoperative serum samples of iPAH and CTEPH patients were obtained 6 to 12 months after surgical interventions. Postoperative AVS serum samples were collected 10 days after surgery. Postoperative sera were not obtained from patients with follow-up treatment at other healthcare facilities or without follow-up treatment. Healthy volunteers donated blood once (Figure 1). Blood samples were incubated at room temperature for 30 min, centrifuged at 2850 relative centrifugal force for 15 min at 4 °C, and cryopreserved <−70 °C.

### 2.4. Analyte Quantifications

Commercially available enzyme-linked immunosorbent assays (ELISA) were used to quantify serum S100A4, EGF, and EGFR in patient cohorts and controls (S100 Calcium Binding Protein A4—ELISA, Uscn. Life Science Inc., Aachen, Germany; Human EGF and human EGFR ELISA, both R&D Systems, Minneapolis, MN, USA). ELISAs were performed according to the manufacturers’ instructions and analyte concentrations were determined using external standards.

### 2.5. Tissue Sample Collection and Immunohistochemistry (IHC)

In our study, 29 cases of histologically proven iPAH and 10 CTEPH cases were histopathologically investigated by the Department of Pathology, Medical University of Vienna. Proximal specimens were collected during PEA. In parallel, samples were obtained from resected lungs of iPAH patients. Samples were formalin-fixed, paraffin-embedded, cut into serial sections of 2–5 μm thickness, and kept refrigerated until further processing.

Microsections were incubated at 50 °C for one hour and deparaffinized using 2 × Histo SAV (SAV Liquid Production GmbH, Flintsbach am Inn, Germany), propanol, ethanol (2 × 100%, 2 96%, 1 × 80%, 1 × 70%, and 1 × 50%), and distilled water. For antigen retrieval, slides were heated in a citrate buffer (Target Retrieval Solution, Dako REAL, Agilent, Santa Clara, CA, USA) (3 × 450 Watt for 5 min). Samples were subjected to 0.3% hydrogen peroxide to quench endogenous peroxidase. Slides were incubated with blocking serum (Vectastain ABC Kit, rabbit IgG, Vector Laboratories, Burlingame, CA, USA) and primary antibodies directed against human S100A4 (Abcam, rabbit polyconal IgG antibody to human S100A4, dilution: 1:200, Cambridge, UK) at 4 °C overnight. After washing, biotinylated anti-rabbit secondary antibody and avidin-peroxidase conjugates (both Vectastain) were added. Peroxidase activity was detected by 3,3′-Diaminobenzidine (DAB tablets, SIGMAFAST, Sigma Aldrich, St. Louis, MO, USA). Nuclei were visualized by Mayers’ hematoxylin. Samples were dehydrated by serial ethanol concentrations (1 × 70%, 1 × 96%, 1 × 100%), n-butyl, and xylene. Lastly, slides were mounted in resinous medium (Pertex, neutral xylene-soluble mounting medium, Medite Medizintechnik, Burgdorf, Germany).

EGFR staining was performed by autostaining using CONFIRM anti-EGFR antibody (clone 3C6, Ventana Medical Systems, Oro Valley, AZ, USA).

### 2.6. Statistical Methods

SPSS Statistics software version 23 (IBM SPSS Inc., Chicago, IL, USA) was used for statistical analysis. GraphPad Prism 5 (GraphPad Software, La Jolla, CA, USA) was used for graphical data presentation. Normal distribution of data was determined by graphical visualization. Differences between two independent groups without normal distribution were determined by Mann–Whitney U test. Wilcoxon signed-rank test was used for longitudinal analysis of pre- and post-operative variables. Chi-square test was used to analyze independent dichotomous variables. One-way ANOVA with Bonferroni–Holm correction was used to compare three groups. *p*-values < 0.05 (two-tailed) were considered statistically significant and data are presented as mean ± standard deviation.

## 3. Results

### 3.1. CTEPH and iPAH Display Decreased Serum S100A4 Levels Compared to AVS and Healthy Individuals

**Figure 2:**
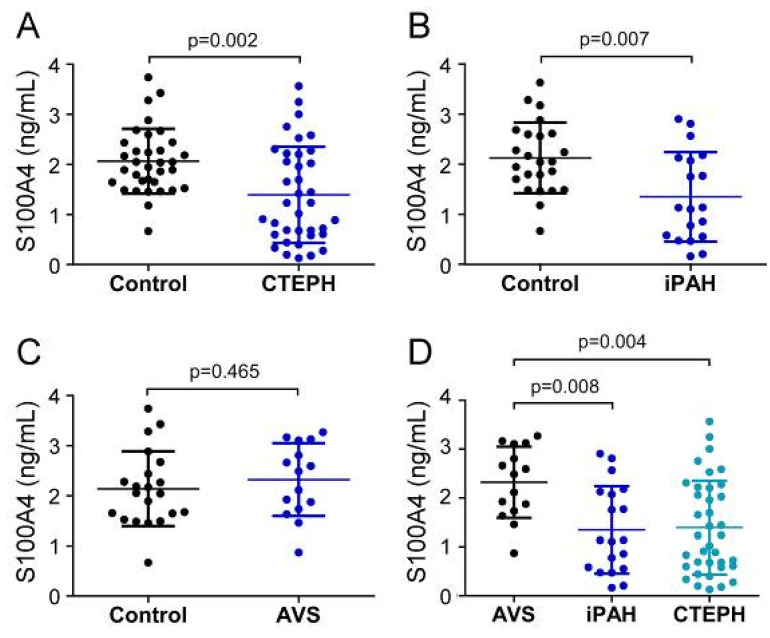
Serum concentrations of S100A4 in (**A**) CTEPH, (**B**) iPAH, and (**C**) AVS patient groups and controls. (**D**) Comparison of S100A4 levels between AVS, iPAH, and CTEPH patients. Each dot represents one donor. Arithmetic means and standard deviations are indicated by horizontal lines. Patient groups were compared to controls by Mann–Whitney U test. One-way ANOVA with Bonferroni–Holm post hoc test was used to compare 3 groups.

First, we compared the pre-surgery status of serum EndMT markers between patient groups and allocated healthy controls. Before surgery, systemic S100A4 levels of CTEPH patients (1394.4 ± 960.4 pg/mL) and iPAH (1349.3 ± 894.6 pg/mL) were significantly decreased compared to healthy controls (CTEPH-control: 2065.6 ± 648.4 pg/mL; *p* = 0.002; iPAH-control: 2127.5 ± 706.8 pg/mL; *p* = 0.007) (Figure 2A,B). In contrast, S100A4 concentrations in AVS patients (2320.6 ± 727.2 pg/mL) showed no difference compared to controls (2138.5 ± 745.9 pg/mL; *p* = 0.465) (Figure 2C). One-way ANOVA revealed a significant downregulation of S100A4 in iPAH and CTEPH patients compared to AVS (iPAH: *p* = 0.008 and CTEPH: *p* = 0.004, respectively) (Figure 2D).

### 3.2. Elevated EGF Concentration in CTEPH Compared to AVS and Controls

**Figure 3:**
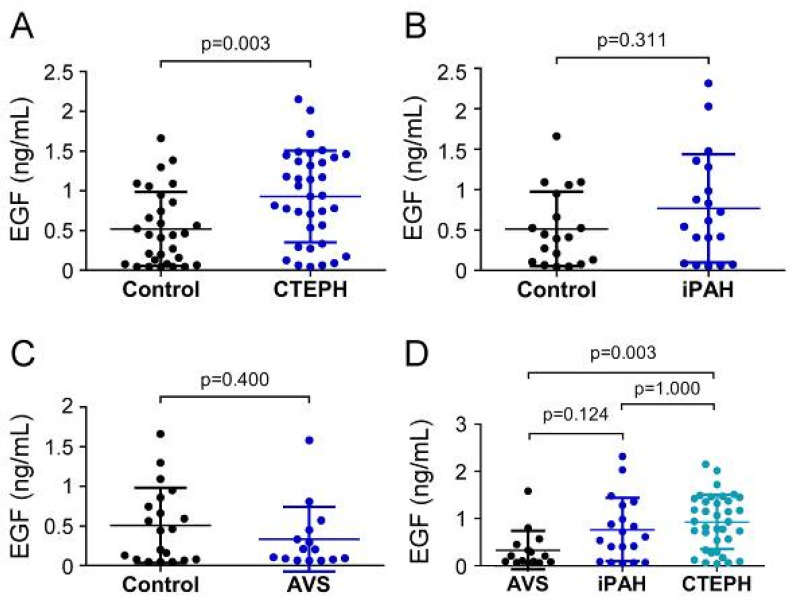
Serum concentrations of EGF in (**A**) CTEPH, (**B**) iPAH, and (**C**) AVS patient groups and controls. (**D**) Comparison of EGF levels between AVS, iPAH, and CTEPH patients. Each dot represents one donor. Arithmetic means and standard deviations are indicated by horizontal lines. Patient groups were compared to controls by Mann–Whitney U test. One-way ANOVA with Bonferroni–Holm post hoc test was used to compare 3 groups.

EGF serum concentrations in CTEPH patients (926.61 ± 576.61 pg/mL) were remarkably increased compared to controls (520.0 ± 464.2 pg/mL; *p* = 0.003) (Figure 3A), while EGF concentrations (766.0 ± 669.9 pg/mL) displayed no difference in the iPAH group compared to controls (511.9 ± 460.3 pg/mL; *p* = 0.311) (Figure 3B). Comparably, preoperative serum concentrations of EGF in the AVS group (331.3 ± 408.0 pg/mL) were not different from controls (506.5 ± 473.5 pg/mL; *p* = 0.400) (Figure 3C). EGF concentrations of CTEPH patients were significantly higher than the AVS group (*p* = 0.003) (Figure 3D). The iPAH group showed slightly yet non-significantly elevated EGF concentrations compared to the AVS group (*p* = 0.124).

### 3.3. Downregulation of Systemic EGFR Levels in CTEPH, iPAH, and AVS Compared to Controls

**Figure 4:**
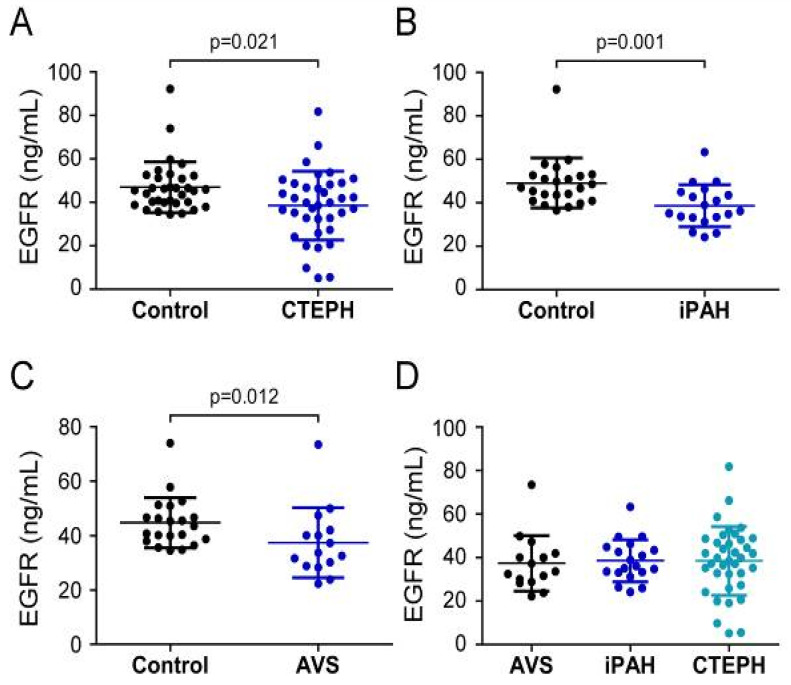
Serum concentrations of EGFR in (**A**) CTEPH, (**B**) iPAH, and (**C**) AVS patient groups and controls. (**D**) Comparison of EGFR levels between AVS, iPAH, and CTEPH patients. Each dot represents one donor. Arithmetic means and standard deviations are indicated by horizontal lines. Patient groups were compared to controls by Mann–Whitney U test. One-way ANOVA with Bonferroni–Holm post hoc test was used to compare 3 groups.

Analysis of EGFR serum concentrations showed that CTEPH patients (38.52 ± 15.81 ng/mL) and iPAH patients (38.6 ± 9.7 ng/mL) displayed lower values when compared to healthy volunteers (CTEPH controls 47.1 ± 11.6 ng/mL, *p* = 0.021; iPAH controls 49.1 ± 11.4 ng/mL, *p* = 0.001) (Figure 4A,B). In contrast to S100A4 and EGF, preoperative EGFR levels in AVS patients (37.4 ± 12.8 ng/mL) were decreased compared to controls (44.8 ± 9.2 ng/mL; *p* = 0.012) (Figure 4C). One-way ANOVA of preoperative EGFR concentrations of PH and AVS patients showed no difference (*p* = 0.962) (Figure 4D).

### 3.4. Serum Analyte Concentrations Remain Unaltered by Surgical Interventions

**Figure 5:**
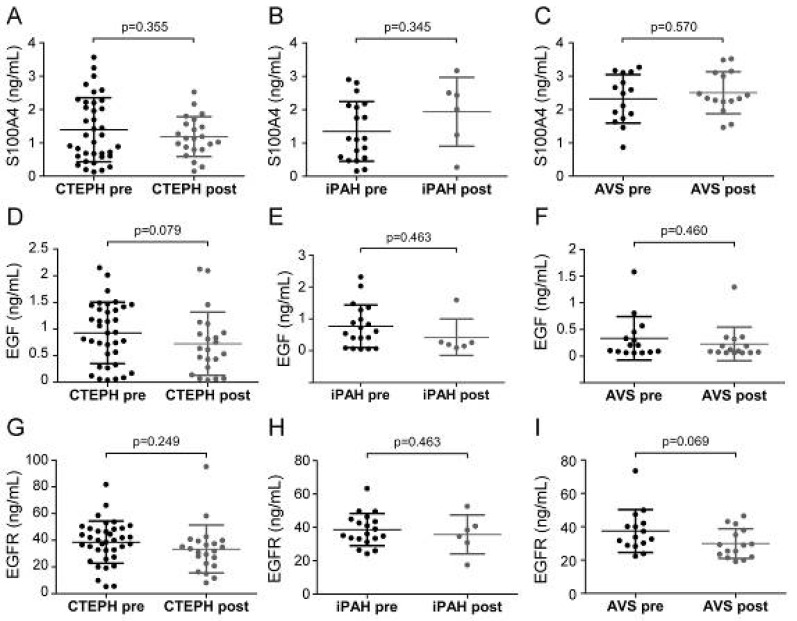
Pre- and post-operative serum concentrations of S100A4, EGF, and EGFR. Pre- and post-surgical S100A4 levels were determined for (**A**) CTEPH, (**B**) iPAH, and (**C**) AVS. EGF concentrations before and after surgery in (**D**) CTEPH, (**E**) iPAH, and (**F**) AVS patients. EGFR levels of (**G**) CTEPH, (**H**) iPAH, and (**I**) AVS patients before surgery and after follow-up. Longitudinal comparisons were performed by Wilcoxon signed-rank test. Each dot represents one patient. Arithmetic means and standard deviations are indicated by horizontal lines.

Next, we sought to determine whether surgical therapies affected serum EndMT marker levels. Serum samples at preoperative and postoperative time points were available for 23 CTEPH, 7 iPAH, and 15 AVS patients. Patient conditions displayed pronounced improvement, evidenced by higher physical performance, reduced dyspnea, increased cardiac index, and decreased meanPAP. Longitudinal analyses revealed no difference of serum EndMT levels between pre- and post-operative time points (Figure 5).

### 3.5. S100A4 Expression in Lung Tissue Positively Correlates with Advanced Heath–Edwards Stages

**Figure 6:**
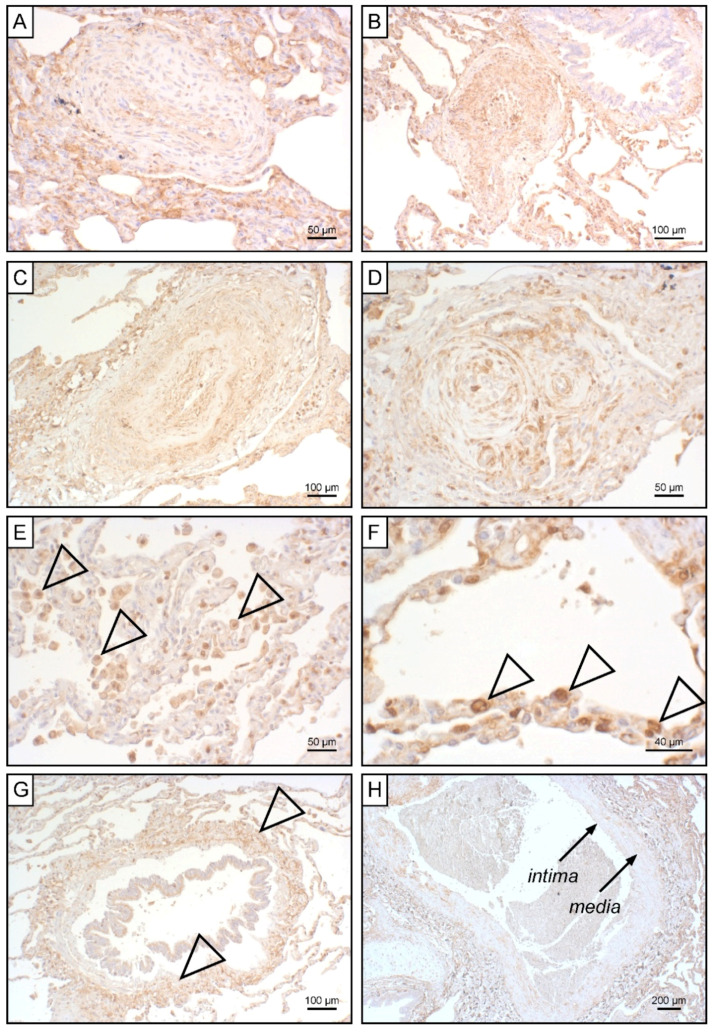
S100A4 expression in iPAH patients. Immunohistochemical staining of S100A4 in iPAH lung tissues. Representative micrographs of characteristic pulmonary artery changes in small vessels according to Heath–Edwards classification (**A**) grade I, (**B**) grade II, (**C**) grade III, and (**D**) grade IV are shown. (**E**) Alveolar macrophages, (**F**) alveolar epithelium, (**G**) peribronchiolar fibrous tissue, and (**H**) tunica intima and tunica media of larger pulmonary vessels positive for S100A4 are shown. Tissues of interest are highlighted by open arrowheads.

**Figure 7:**
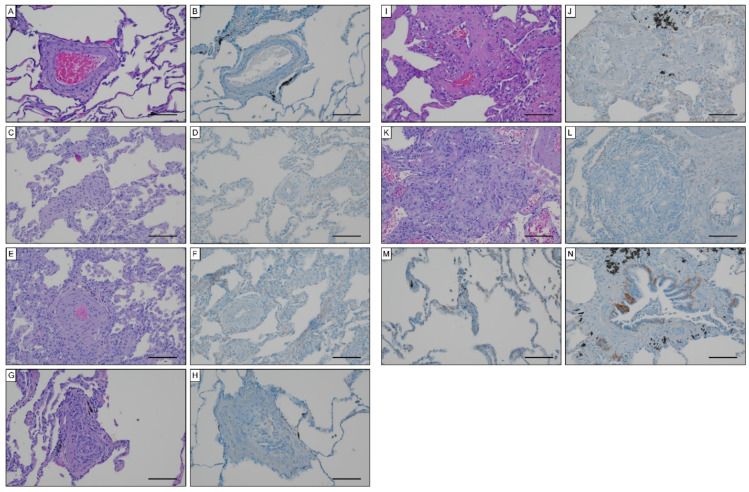
EGFR expression in iPAH specimen. Hematoxylin eosin and EGFR staining in (**A**,**B**) Heath–Edwards grade I, (**C**,**D**) grade II, (**E**,**F**) grade III, (**G**,**H**) grade IV, (**I**,**J**) grade V lesions, (**K**,**L**) plexiform lesions. EGFR staining of (**M**) alveolar pneumocytes and (**N**) basal epithelium of bronchioles. Scale bars, 100 µm.

To unravel potential sources for systemic EndMT markers, we determined tissue expression of S100A4 and EGFR in iPAH lungs and endarterectomized tissues obtained from CTEPH patients. Grade I Heath–Edwards pathohistological lesions were observed in 13 (44.8%) specimens, of which 4 (30.8%) displayed positive S100A4 staining (Figure 6A, Table 4). All (100%) higher grade lesions (≥grade II) in iPAH lungs stained positive for S100A4 (Figure 6B–D). Alveolar macrophages (Figure 6E) and epithelium (Figure 6F) expressed S100A4. Peribronchial fibrous tissue showed S100A4 staining in 96.4% of all cases (Figure 6G, Table 4), while perivascular tissue was devoid of S100A4 expression. In larger vessels, S100A4 positive intima and media were observed in 81.0% and 57.1% of all cases, respectively (Figure 6H). S100A4 and EGFR were not detectable in lung tissues of CTEPH patients (data not shown). Similarly, EGFR was not found in iPAH lesions of varying grades (Figure 7A–L). However, we observed EGFR immunoreactivity in pneumocytes of alveoli (Figure 7M) and the basal epithelium of bronchioles (Figure 7N).

## 4. Discussion

In the current study, we were able to demonstrate a distinct regulation of systemic and local EndMT factors in PH with RVH. We observed a PH-specific downregulation of S100A4 and EGFR in CTEPH and in iPAH. While EGF levels of iPAH patients were comparable to controls, CTEPH displayed increased serum EGF. Intriguingly, systemic EndMT factors remained unaltered by surgical interventions. Locally, CTEPH lung specimen were devoid of S100A4 and EGFR. Lung parenchyma of iPAH patients displayed S100A4 staining in higher-grade pathological lesions, while EGFR was not detectable.

In PH pathology, substantial architectural and functional changes occur in the pulmonary vasculature. Though several underlying and perpetuating pathomechanistic events have already been identified, the picture of PH vasculopathy remains complex and incomplete. The alterations vary in different types of PH but usually involve arterial and venous restructuring. Several cell types of the lung, such as endothelial, mesenchymal, and smooth muscle cells, are involved in disease onset and progression. Originally, vascular smooth muscle cells (vSMCs) were considered the exclusive origin of alpha smooth muscle actin (αSMA)-expressing mesenchymal cells in PAH. Since then, several studies were able to demonstrate that pulmonary arteriolar endothelial cells give rise to SMC-like cells during hypoxia-induced pulmonary vascular remodeling [18,33,34]. Pulmonary endothelial cells promote inflammation and activation of adjacent SMCs resulting in pulmonary fibrosis and chronic obstruction of the affected vessels, indicating that dysregulated mesenchymal cells play a key role in the development of CTEPH [35]. In CTEPH, myofibroblast-like cells of the vascular clot induced cellular elongation and loss of cell–cell contacts in endothelial cells [36], suggesting mesenchymal cells as a contributing factor to the transition of endothelial cells. A previous work reported that endothelial cells and smooth muscle cells in pulmonary arteries of CTEPH patients obtained a migratory and proliferative phenotype in vitro [37]. These findings were extended by a study where migratory endothelial cells with a mixed mesenchymal/endothelial phenotype were detected in PAH lungs in situ [21]. An in vivo study showed that hyperproliferative, invasive myofibroblast-like cells from endarterectomized tissue of CTEPH patients were prone to develop tumors at the intimal pulmonary vessel layer in mice [38]. These data suggest that PH and the cellular microenvironment promote the conversion of healthy endothelial cells into dysfunctional mesenchymal cells, which, in turn, aggravate disease symptomatology and might serve as a potential source for systemic and local EndMT markers.

Various lung pathologies, such as plexogenic arteriopathy [39], chronic obstructive pulmonary disease [40], and several neoplastic conditions [41,42], have been associated with increased S100A4 levels and poor prognosis [43,44]. Interestingly, S100A4 has further been implicated in PH development and RVH [31], and increased S100A4 levels have been reported in the crosstalk between macrophages and pulmonary fibroblasts to promote pulmonary fibrosis [45,46]. Moreover, S100A4 is considered a crucial factor in EndMT [47,48]. Ambartsumian and colleagues reported that approximately 5% of mice ubiquitously overexpressing S100A4/*Mts1* develop pulmonary artery changes resembling plexogenic lesions [49]. Intriguingly, Greenway et al. found a positive correlation between S100A4 levels and severity of vascular lesions in human plexogenic arteriopathy. The authors thus postulated that S100A4 was not implicated in the initial response to pulmonary hypertension but might exhibit functional relevance in advanced stages [39]. These data are corroborated by our histological findings where elevated S100A4 expression was detected in higher-grade pathological lesions. In detail, Greenway et al. reported S100A4 expression in neo-intimal smooth muscle cells, which is in accordance with our observation of S100A4 expression in the intima of resected iPAH lungs. Though intimal staining prevailed, we were further able to detect medial S100A4 expression. Our finding of S100A4^+^ alveolar macrophages is in line with data reported by Greenway et al. who described S100A4/Mts1^+^ periadventitial cells in mice with occlusive neointimal lesions. Together, these data indicate a role for S100A4 in PH initiation, disease progression, and the local inflammatory milieu. Delineating the exact contribution of S100A4 to the pathomechanistic events driving PH-associated vasculature remodeling merits future studies.

While the majority of previous studies was confined to investigating the local S100A4 status, we assessed S100A4 on a systemic level. Our quantifications of serum S100A4 in healthy controls are in line with a previous report of plasma S100A4 by Peng and colleagues [50]. However, Peng et al. observed strongly elevated plasma S100A4 in patients with portopulmonary hypertension, a subtype of group 1 PH with portal hypertension. In contrast to these reports, we detected decreased serum S100A4 in CTEPH and iPAH and S100A4 levels displayed a tendency towards further decrease following surgical intervention and recovery. S100A4 can be secreted and exerts paracrine, extracellular functions [51]. Interestingly, Schneider et al. observed that cardiac myocytes from patients with ischemic cardiomyopathy and aortic stenosis stained positive for S100A4 but were devoid of *S100A4* mRNA [52]. The authors thus surmised that cardiomyocytes take up or bind S100A4 released from other cells rather than synthesizing S100A4 themselves. This finding might provide a potential explanation for the diminished S100A4 serum levels detected in our study groups. The mechanisms perpetuating S100A4 secretion might become exhausted in the more advanced stages of PH, the remaining S100A4 being resorbed by target tissues. Further investigations will be required to determine the origin and destination of circulating S100A4 in PH. Furthermore, S100A4 tissue levels seem to be strongly associated with disease stage. In our study, we mainly enrolled patients with end stage PH. Hence, the EndMT dynamics detected here might no longer resemble the status at disease onset or during disease progression. As EndMT might play distinct roles in disease onset, progression, and end stage, more elaborate studies are necessary to capture the dynamic turnover of systemic and local EndMT factors in the course of PH.

EGFR is overexpressed in several cancers and dysregulated EGFR signaling contributes to cancer metastasis and progression by promoting epithelial-to-mesenchymal transition [53]. Though the EGF/EGFR axis is implicated in EndMT [20], systemic EGFR was downregulated in CTEPH, in iPAH, and in AVS compared to healthy controls. Conversely, systemic EGF levels remained unaltered in iPAH and AVS but were elevated in CTEPH. Interestingly, intravenous administration of EGF induced medial thickening and increased mPAP [54]. Increased EGF levels thus might contribute to the deterioration of CTEPH pathology.

EGFR expression in pneumocytes and basal epithelial cells of the bronchioles has been shown previously [55]. In addition, only weak immunoreactivity was found in plexiform lesions of iPAH patients [55]. These data are in line with our observation of high EGFR levels in pneumocytes and the basal bronchial epithelium but no EGFR expression in pulmonary lesions. Increased expression of EGFR by pulmonary arterial smooth muscle cells was reported in PH rat models [56], and several experimental studies have already demonstrated a beneficial therapeutic effect of EGFR blockade in PH. Oral administration of an EGFR inhibitor induced regression of PAH in rats [57], subcutaneous injection of a pan EGFR inhibitor attenuated pulmonary vascular remodeling [56], and EGFR antagonists reduced RVH in experimental PAH [58]. Intriguingly, the total levels of EGFR in PH lungs were not different from healthy controls. However, EGFR auto-phosphorylation was increased in the pulmonary artery wall of patients presenting with grade 5 Heath–Edwards classification compared to controls [59]. As we observed decreased systemic EGFR levels in our groups compared to controls and no EGFR expression in the pulmonary vasculature, future studies are warranted to determine the underlying mechanisms of inhibiting EGFR to improve PH.

In order to determine whether the regulation of EndMT marker is specific for right heart disease, we compared the parameters between PH patients suffering from RVH with those of AVS patients suffering from left heart disease. Serum S100A4 levels were downregulated in PH, while showing no difference to controls in AVS. EGF was elevated in CTEPH but were not different from controls in iPAH and AVS. By comparison, EGFR was diminished in all groups investigated when compared to healthy individuals. Though several studies demonstrated a role for EGFR-dependent signaling in cardiac hypertrophy and EGFR inhibitors attenuated organ deterioration [60,61,62], our findings indicate that systemic EGF and EGFR display no specificity for PH and RVH. Of note, our data revealed that systemic downregulation of S100A4 was PH- and RVH-specific. Intriguingly, our finding of decreased systemic S100A4 is in contrast to previous reports, where elevated S100A4 tissue levels were associated with cardiac hypertrophy. S100A4 expression in cardiomyocytes of patients suffering from hypertrophic cardiomyopathy was increased [63], and S100A4 was found to be upregulated following transition to heart failure [64]. In addition, S100A4 was induced by acute ischemia and aortic stenosis and exerted pro-hypertrophic as well as cardio-protective effects [52]. These data indicate that local and systemic S100A4 in cardiac hypertrophy display opposing tendencies. Determining the potential relevance of decreased serum S100A4 in PH patients presenting with RVH remains the subject of future investigations.

Though clinical and hemodynamic parameters improved by PEA, LuTX, and AVR, pre- and post-operative serum EndMT marker levels displayed no differences. This observation is in line with our previous work where sRAGE remained unaltered by PEA, LuTX, and AVR [29]. These data indicate that surgical therapy does not affect systemic EndMT marker and that systemic levels do not reflect the prevailing disease stage.

### Limitations

In spite of our best efforts, we recognize some limitations of this work. We studied a limited number of patient samples and investigating larger patient cohorts might reveal further differences and alterations in EndMT marker levels that we were not able to be captured here. Furthermore, investigating larger patient numbers allows for further patient stratification according to other characteristics, such as mortality, disease severity, and comorbidities. In our study, only operable patients were included. Patients considered not suitable to undergo surgical therapy but receiving medical therapy or rehabilitation instead were not tested. Future studies are required to compare EndMT markers between operable patients and non-surgical patients. Another limitation of our study is that patients were treated with different surgical approaches. While PEA on a cardiopulmonary bypass was performed for CTEPH patients, LuTX on extracorporeal membrane oxygenation was carried out for iPAH patients. Furthermore, the medical therapies, such as immunosuppressive agents following LuTX, differed between groups. An inevitable limitation is the reduced age of iPAH compared to CTEPH and AVS patients. We determined systemic and local EndMT marker concentrations using ELISA and IHC, respectively, and these immunoassays are not validated for clinical laboratory routine. In addition, IHC represents a qualitative rather than a quantitative method and can be subjective. Further research is necessary to elaborate on the exact tissue levels of EndMT markers. For example, quantitative PCR and Western blot analyses are more suitable to perform detailed quantification of RNA and protein levels of EndMT markers in lungs of PH patients. As our data suggest an involvement of S100A4, EGF, and EGFR in PH, the investigation of additional factors implicated in these signaling pathways will be necessary to substantiate our findings.

## 5. Conclusions

Taken together, our data display a heterogeneous picture of EndMT markers in PH. While S100A4 was systemically reduced in patients with PH, the tissue levels were increased in advanced grades of Heath–Edwards lesions. EGR was exclusively increased in CTEPH, but systemic EGFR was decreased in CTEPH and iPAH compared to healthy individuals. Interestingly, EGFR was not detectable in CTEPH lungs and in iPAH pathological lesions. These insights indicate a complex role of EndMT markers in PH pathology and a highly PH subtype-specific regulation of these factors. Furthermore, it seems that systemic levels do not necessarily reflect local tissue expression. This finding indicates that the disease stage cannot be determined by measuring systemic EndMT factors.

In our study, we selected the specific EndMT markers S100A4, EGF, and EGFR. Investigating further conventional EndMT markers, such as TWIST, SNAI1, SNAI2, vascular endothelial cadherin, and CD31, will be the subject of further investigations. In addition, exploring the effect of PH-specific pharmacological therapy on systemic and local EndMT markers might improve our understanding of EndMT in PH pathology. Studying EndMT factors in other cohorts, such as patients suffering from chronic thromboembolic pulmonary disease (CTEPD) with and without PH, might help to shed more light on the role of EndMT in pulmonary diseases.

Future studies are required to determine whether treatment regimens targeting processes involved in EndMT and pulmonary vascular remodeling might represent a promising therapeutic avenue in PH to prevent RVH and to decrease mortality.

## 6. Patents

The authors hold no patents related to this work.

## Figures and Tables

**Table 1:** Basic demographic and hemodynamic data of CTEPH patients. Concentrations of EndMT biomarkers in CTEPH patients and healthy controls are reported. Controls were age- and sex-matched.

	CTEPH	Control	*p*-Value
*n*	37	39	
follow-up (*n*)	23		
age in years *	57.54 (61.00) ± 13.37 [31–78]	52.44 (53) ± 13.16 [31–80]	0.111
F:M ratio [%]	13:24 (35.1:64.9)	15:19 (44.1:55.9)	0.475
initial NYHA ^1^	2.89 (3) ± 0.68 [2–4]		
prePAPmean * [mmHg] ^2^	49.65 (50.5) ± 14.26 [27–90]		
postPAPmean * [mmHg] ^2^	27.33 (27) ± 6.08 [14–41]		<0.0001 ^§^
prePVR * [mmHg × min/L] ^2^	8.48 (8.22) ± 2.89 [3.43–12.74]		
postPVR * [mmHg × min/L] ^2^	2.66 (2.37) ± 1.33 [0.84–4.66]		<0.0001 ^§^
preCI * [L/min/m^2^] ^2^	2.33 (2.22) ± 0.5 [1.59–4.2]		
postCI * [L/min/m^2^] ^2^	3.08 (2.94) ± 0.94 [1.8–6.3]		0.0007 ^§^
S100A4 * [pg/mL] ^3^	1394.44 (1231.8) ± 960.41 [128.37–3564.6]	2065.60 (1992.85) ± 648.39 [668.57–3739.46]	0.002
EGF * [pg/mL] ^3^	926.61 (933.83) ± 576.61 [43.45–2149.12]	520.01 (443.74) ± 464.24 [43.46–1658.42]	0.003
EGFR * [ng/mL] ^3^	38.52 (39.84) ± 15.81 [5.3–81.77]	47.05 (45.52) ± 11.64 [34.54–92.22]	0.021

* Reported are mean (median) ± standard deviation [min–max]. ^1^ NYHA score was determined based on patient symptoms; ^2^ determined by right heart catheterization; ^3^ quantified by commercially available ELISAs. ^§^ comparing pre- versus post-operative values. *CTEPH* chronic thromboembolic pulmonary hypertension, *n* number of patients, *F:M* ratio female to male ratio, *NYHA* New York Heart Association, *prePAPsys* preoperative systolic pulmonary artery pressure, *prePAPdia* preoperative diastolic pulmonary artery pressure, *prePAPmean* preoperative mean pulmonary artery pressure, *postPAPmean* postoperative mean pulmonary artery pressure, *prePVR* preoperative pulmonary vascular resistance, *preCI* preoperative cardiac index, *S100A4* member of S100 family of Ca^+^ binding proteins, *EGF* epidermal growth factor, *EGFR epidermal growth factor receptor*.

**Table 2:** Basic demographic and hemodynamic data of iPAH patients. Several serum parameters of iPAH patients are shown. Concentrations of EndMT biomarkers in iPAH patients and healthy controls are reported. Controls were age- and sex-matched.

	iPAH	Control	*p*-Value
*n*	19	24	
follow-up (*n*)	7		
age in years *	37.84 (35) ± 8.34 [21–54]	43.21 (38.5) ± 13.93 [26–76]	0.126
F:M ratio [%]	15:4 (78.9:21.1)	17:7 (70.8:29.2)	0.728
initial NYHA ^1^	3.85 (4) ± 0.36 [3–4]		
prePAPsys * [mmHg] ^2^	124.69 (120) ± 34.36 [65–180]		
prePAPmean * [mmHg] ^2^	66.25 (66) ± 24.55 [27–101]		
preCI * [L/min/m^2^] ^2^	2.27 (2.3) ± 0.2 [2–2.5]		
normal postPAPsys ^3^	7 of 11 patients (63.6%) ^§^		
creatinine * [mg/dL] ^4^	1.02 (0.99) ± 0.32 [0.5–1.65]		
BUN * [mg/dL] ^4^	24.2 (17.45) ± 18.05 [8.5–73]		
protein serum * [g/L] ^4^	67.29 (69.35) ± 11.6 [39.7–83.3]		
cholinesterase * [k/U] ^4^	4.74 (4.71) ± 1.26 [1.8–6.25]		
S100A4 * [pg/mL] ^5^	1349.27 (1134.4) ± 894.64 [160.9–2905.4]	2127.47 (2045.65) ± 706.82 [668.57–3630.87]	0.007
EGF * [pg/mL] ^5^	766.01 (612.08) ± 669.87 [48.30–2313.62]	511.86 (410.03) ± 460.25 [43.46–1658.42]	0.311
EGFR * [ng/mL] ^5^	38.61 (36.12) ± 9.66 [24.24–63.33]	49.08 (46.96) ±11.42 [36.45–92.22]	0.001

* Reported are mean (median) ± standard deviation [min–max]. ^1^ NYHA score was determined based on patient symptoms; ^2^ determined by right heart catheterization; ^3^ determined by echocardiography; ^4^ determined by validated routine laboratory tests; ^5^ quantified by commercially available ELISAs; ^§^ 7 of 11 patients displayed no signs of tricuspid insufficiency, indicating normal postPAPsys, while elevated postPAPsys was found in 4 patients with 30, 37, 56, and 70 mmHg, respectively. *iPAH idiopathic pulmonary arterial hypertension*, *n* number of patients, *F:M* ratio female to male ratio, pre*PAPsys* preoperative systolic pulmonary artery pressure, *prePAPmean* preoperative mean pulmonary artery pressure, *preCI* preoperative cardiac index, *postPAPsys* postoperative systolic pulmonary artery pressure, *BUN* blood urea nitrogen, *S100A4* member of S100 family of Ca^+^ binding proteins, *EGF* epidermal growth factor, *EGFR epidermal growth factor receptor*.

**Table 3:** Basic demographic and hemodynamic data of AVS patients. Several serum parameters of AVS patients are shown. Concentrations of EndMT biomarkers in AVS patients and healthy controls are reported. Controls were age- and sex-matched.

	AVS	Control	*p*-Value
*n*	15	21	
follow-up (*n*)	15		
age in years *	65.23 (64.5) ± 10.31 [44–86]	60.29 (61) ± 9.47 [38–80]	0.146
F:M ratio [%]	6:9 (40:60)	8:13 (38.1:61.9)	1.000
prePAPmean * [mmHg] ^1^	18.86 (19) ± 4.94 [9–28]		
Vmax * [m/s] ^2^	4.46 (4.3) ± 0.73 [3.8–6.2]		
ejection fraction * [%] ^2^	57.26 (57.00) ± 7.46 [45–73]		
creatinine * [mg/dL] ^3^	1.13 (0.96) ± 0.48 [0.67–2.1]		
cholesterol * [mmol/L] ^3^	4.36 (4.2) ± 0.99 [3–7.1]		
triglyceride * [mmol/L] ^3^	1.82 (1.5) ± 1.15 [0.69–4.9]		
S100A4 * [pg/mL] ^4^	2320.63 (2485.75) ± 727.22 [868.33–3268.11]	2138.51 (2053) ± 745.94 [668.57–3739.46]	0.465
EGF * [pg/mL] ^4^	331.32 (203.91) ± 408.01 [60.37–1577.63]	506.45 (451.79) ± 473.49 [44.49–1658.42]	0.400
EGFR * [ng/mL] ^4^	37.43 (33.67) ± 12.77 [22.27–73.45]	44.76 (43.57) ± 9.18 [34.54–73.93]	0.012

* Reported are mean (median) ± standard deviation [min–max]. ^1^ determined by right heart catheterization; ^2^ determined by echocardiography; ^3^ determined by validated routine laboratory tests; ^4^ quantified by commercially available ELISAs. *AVS aortic valve stenosis*, *n* number of patients, *F:M* ratio female to male ratio, pre*PAPmean* preoperative mean pulmonary artery pressure, *S100A4* member of S100 family of Ca^+^ binding proteins, *EGF* epidermal growth factor, *EGFR epidermal growth factor receptor. Vmax peak jet velocity*.

**Table 4:** S100A4 expression Heath–Edwards lesions and iPAH lungs (*n* = 29). Pathological vascular lesions classified according to Heath–Edwards [9,10] as well as perivascular and peribronchial expression were analyzed.

	Cases per Group (%)	Numbers of Positively Stained Cases per Group (%)
grade I lesions	13 (44.8)	4 (30.8)
grade II lesions	17 (58.6)	17 (100)
grade III lesions	8 (27.6)	8 (100)
grade IV+ lesions	8 (27.6)	8 (100)
peribronchial staining	28 (96.6)	27 (96.4)
perivascular staining	27 (93.1)	0 (0)
intima staining in larger vessels	21 (72.4)	17 (81)
media staining in larger vessels	21 (72.4)	12 (57.1)

## Data Availability

Raw data are available upon request.
